# Comparison of periplasmic and intracellular expression of *Arabidopsis* thionin proproteins in *E. coli*

**DOI:** 10.1007/s10529-013-1180-z

**Published:** 2013-03-21

**Authors:** Amjad Abbas, Stephan Plattner, Kausar Hussain Shah, Holger Bohlmann

**Affiliations:** Division of Plant Protection, Department of Crop Sciences, University of Natural Resources and Life Sciences, Vienna, UFT Tulln, Konrad Lorenz Str. 24, 3430 Tulln, Austria

**Keywords:** Fusion protein, His-tag, Maltose-binding protein, Tobacco etch virus, Thionin, Thioredoxin

## Abstract

Thionins are antimicrobial plant peptides produced as preproproteins consisting of a signal peptide, the thionin domain, and a so-called acidic domain. Only thionin itself has been isolated from plants. To study the processing of the precursor, it has to be produced in a heterologous system. Since both domains contain several cysteines and, due to the known antimicrobial activity of the thionin, we tested the expression of all four *Arabidopsis* proproteins as fusion proteins. Periplasmic expression as fusion with maltose binding protein was not successful but cytoplasmic expression as His-tagged TRX fusion proteins with a TEV recognition sequence resulted in proteins of correct size. Use of the SHuffle strain C3030 further improved the expression. Fusion proteins inhibited growth of *Escherichia coli*. They could be cleaved by TEV protease, releasing authentic proproteins without any additional amino acid at the *N*-terminus.

**Electronic supplementary material:**

The online version of this article (doi:10.1007/s10529-013-1180-z) contains supplementary material, which is available to authorized users.

## Introduction

Thionins are plant antimicrobial peptides (AMP) that are generally basic. They contain approximately 45 amino acids and 6 or 8 cysteine residues which form disulfide bridges (see Bohlmann 1994). Their general structure is that of two small α-helices and two small antiparallel β-sheets stabilized by three or four disulfide bridges (Stec 2006). Their toxicity and antimicrobial activity seem to depend on the destruction of membranes.

Thionins have been isolated as 5 kDa peptides from various plant species but they are synthesized as much larger preproproteins (Ponz et al. 1986). This has been confirmed by the cloning of different thionin cDNAs and genomic clones showing that all precursors consist of a typical *N*-terminal signal sequence, the thionin itself, and a *C*-terminal acidic domain. Most acidic domains have six cysteine residues (Fig. S1) and are usually acidic (as the name indicates). A protein corresponding to an acidic domain of a thionin precursor has never been isolated from plants. To date, there is no experimental information available about possible functions of the acidic domain.

The model plant, *Arabidopsis* *thaliana,* contains four thionin genes that are expressed in different tissues and organs of the plant. To test the antimicrobial activity in vitro it will be necessary either to isolate the peptides from *Arabidopsis* or to produce them in an expression system. Isolating the thionins from *Arabidopsis* would require large amounts of material. Furthermore, thionin proproteins and acidic domain peptides might be directly processed after synthesis and have not been isolated from any plant species. We were therefore interested to produce thionin proproteins in *Escherichia coli*. Since previous observations (H Bohlmann unpublished results) indicated that the proproteins also might have antimicrobial activity, we decided to produce them as fusion proteins. Thionins and the acidic domain contain several cysteine residues and disulfide bridges are not easily formed within the *E. coli* cytoplasm. We, therefore, first tested the expression of the proproteins as fusions with the maltose-binding protein (MBP) by secretion into the periplasm. We compared this to the expression as thioredoxin (TRX) fusion proteins that are produced in the cytoplasm.

## Methods

### *E. coli* strains (Supplementary Table 1)

We used the DH10B strain for cloning. For protein expression, constructs in pJOE-SP-MCS vectors were transformed into Rosetta (Novy et al. 2001) while pETtrx_1a derived vector constructs were expressed in Rosetta(DE3)pLysS (Novy et al. 2001) and the SHuffle strain C3030 (Lobstein et al. 2012).

### Protein quantification

Proteins were quantified with the Pierce BCA protein assay kit. Protein concentration was measured at 562 nm with a micro-plate reader using BSA as standard.

### Digestion with tobacco etch virus protease (TEV)

We used a mutated TEV protease (TEV_SH_) which was expressed and purified as described by (van den Berg et al. (2006). Purified fusion proteins were incubated with TEV protease at 30 °C for 16 h to release *C*-terminal thionin proproteins (for sequences see Supplementary Fig. 1). To test the effect of reducing agents on digestion efficiency, DTT (at 1 mM) or reduced glutathione/oxidized glutathione (at 3 and 0.3 mM, respectively) were added to the standard digestion buffer (50 mM Tris/HCl, pH 8.0, 0.5 mM EDTA).

### Gel electrophoresis

For fusion of MBPs, polyacrylamide gels (10 % v/v separating and 4 % v/v stacking gel) were used. TRX-fusion protein samples were resolved on Tricine/SDS polyacrylamide gels (16 % v/v separating and 4 % v/v stacking gel) (Schägger 2006).

### Western blotting

A 16 % (v/v) Tricine/SDS gel was run along with a prestained marker (Thermo Fisher Scientific) and was transferred to a Protran-nitrocellulose membrane (Schleicher and Schuell BioSciences, Germany). The transfer time for 25 kDa peptides was 20 min at 10 V. After transfer the membrane was incubated with an rabbit anti-6HIS epitope tag antibody (Rockland, USA) at 1:3000 dilution. Anti-rabbit IgG antibody (Vector Laboratories, Inc) at 1:2000 dilution was used as secondary antibody. Immunoreactive proteins were visualized by reaction with 5-bromo-4-chloro-3-indolyl phosphate/nitro-blue tetrazolium.

### Activity test

Purified (TRX)-fusion proteins were dialysed against water using a dialysis membrane with a MW cut-off of 10,000. *E. coli* Rosetta strain was grown in LB broth. In 96 well microtitre plates, 150 μl cells at OD_600_ of 0.05 were combined with 50 μl protein. Final concentrations of fusion proteins in growth medium were 100, 50, 25, 12.5, 6.25 and 3.125 μg/ml. Kanamycin at 50 μg/ml was used as a positive control while 50 μl water was used as negative control. The plate was incubated at 37 °C in a plate-reader with occasional shaking for 15 s and OD_600_ measurement every 30 min for 8 h.

## Results

### Periplasmic expression

**Fig. 1:**
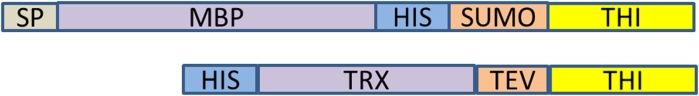
Schematic diagrams of the expression cassettes for thionin proproteins. Top, pJOE-SP-MCS for expression in the periplasm of *E.coli*. *Bottom*, Plasmid pETtrx-1a for expression in the cytoplasm of *E.coli*. *HIS* his-tag; *MBP* maltose binding protein, *SP* signal peptide, *SUMO* small ubiquitin related modifier, *TEV* TEV protease recognition sequence, *THI* thionin proprotein, *TRX* thioredoxin

For the expression of thionin proproteins we wanted to use periplasmic expression since this can produce reasonable amounts of fusion proteins as fusions with protein A (Epple et al. 1997) or MBP (Romero et al. 1997). Protein A has the disadvantage that it binds immunoglobulins and we therefore decided to use MBP as a fusion partner. The plasmid pJOE4905.1 contains a rhamnose-inducible promoter which is tightly regulated (Motejadded and Altenbuchner 2009). It contains the *MalE* gene coding for MBP as a fusion partner. Six histidines for affinity purification are located between the MBP and a codon optimised small ubiquitin-related modifier (SUMO) domain, which provides a recognition site for SUMO protease. For periplasmic expression we introduced a signal peptide (Fikes et al. 1987) as described in Supplementary methods. Furthermore, we introduced a small polylinker which allowed us to use *Bam*HI after the stop codon (Supplementary Fig. 2). We fused the proproteins to the SUMO domain without any linker such that after cleavage no additional amino acid would remain at the *N*-terminus of the proprotein (Fig. 1). All coding sequences for the *Arabidopsis* thionin proproteins were introduced into this vector and confirmed by sequencing.

In all cases a 58 kDa protein was produced after induction while the expected size was 65 kDa (Supplementary Table 2). Periplasmic proteins were isolated by an osmotic shock procedure, but 65 kDa proteins were clearly visible for proTHI2.1 only while a very faint band for proTHI2.2 was detected. The majority of the fusion proteins remained in the insoluble fraction (Supplementary Fig. 3, lanes 4). From the periplasmic protein extract the fusion proteins were enriched by Ni–NTA affinity chromatography but only part of the fusion proteins was recovered as the fusion proteins were also detected in the flow through of the column (Supplementary Fig. 3, lanes 5), perhaps due to weak binding of the His-tag which is located in the middle of the fusion protein. Loss of fusion protein in the flow-through was reported by Motejadded and Altenbuchner (2009). In addition, a large part of the fusion protein was insoluble. We did not test if this fraction remained in the cytoplasm or/and formed inclusion bodies in the periplasm. The only fusion protein that was expressed in larger amounts was the proTHI2.1 fusion protein. The highest yield (Table 1) was obtained for proTHI2.1 (1.4 mg/l). However, it has to be considered that the largest part of these protein fractions will be the 58 kDa protein. A Western blot of the purified protein fractions (Supplementary Fig. 4) also showed that only for proTHI2.1 a fusion protein of the expected size was obtained, although in a very small amount. In all fractions the 58 kDa band was clearly visible on the Coomassie stained gel but was not labeled by the antibody. Instead, the antibody very strongly labeled a 39 kDa band which was not visible by Coomassie staining. We did not try to determine the nature of the 58 and the 39 kDa proteins. Since insufficient amounts of fusion proteins were obtained, we also did not test cleavage of the fusion proteins with SUMO protease.

**Table 1:** Quantification of fusion proteins

Proprotein	MBP—fusion	TRXfusion	
	Rosetta(DE3)pLysS	Rosetta(DE3)pLysS	SHuffle C3030
proTHI2.1	1.4	3.1	3.7
proTHI2.2	0.5	0.7	3.4
proTHI2.3	0.3	2.3	4
proTHI2.4	0.2	1.1	2.8

Fusion proteins were purified from 1 l of culture by Ni–NTA affinity chromatography Protein concentrations (all as mg/l) were determined using BCA test

### Cytoplasmic expression

The periplasmic expression of the thionin proprotein fusion proteins resulted in very low amounts of fusion proteins which mostly did not seem to be of the correct size and was abandoned. We therefore tested the expression of all 4 thionin proproteins as fusions to TRX (Bogomolovas et al. 2009) in the vector pETtrx_1a (Fig. 1). All constructs were transformed into the Rosetta(DE3)pLysS strain. After induction with IPTG strong extra bands of the expected size (25–26 kDa, Supplementary Table 3) were detected on tricine-SDS gels (Supplementary Fig. 5). However, a large part of the fusion proteins was again insoluble. After lysis and centrifugation, the largest amount of the fusion proteins was found in the pellets in all cases (Supplementary Fig. 5, lanes 3 and 4). Ni–NTA chromatography gave higher yields of the fusion proteins as compared to periplasmic expression with proTHI2.1 having the highest yield as compared to the other thionin proproteins (Table 1). We separated all four purified TRX fusion proteins side-by-side on one gel and used an antibody against the His-tag to detect the fusion proteins on a Western blot (Supplementary Fig. 6). The proTHI2.2 fusion showed several small bands at around 15, 14, and 10 kDa that were not seen with the other fusion proteins. These were also labeled with the anti-His-tag antibody and seemed to be degradation products. Western blotting with the anti-His-tag antibody also detected a large band in the proTHI2.1 fraction at around 60 kDa and a smaller (20 kDa) and larger band (30 kDa) in the proTHI2.3 fraction.

**Fig. 2:**
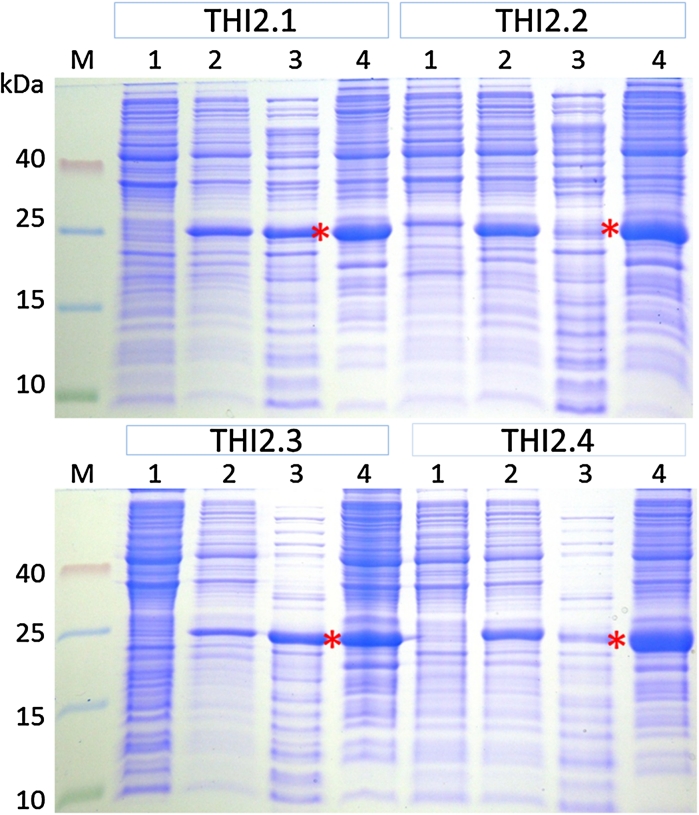
Cytoplamic expression of proTHI-TRX fusion proteins in strain C3030. For all fusion proteins, 1 ml of cell culture was pelleted and dissolved in 100 μl sample buffer. 10 μl from this extract and an equivalent amount for total soluble cytoplasmic fractions were separated on Tricine/SDS gels. (M) Protein marker, (*1*) un-induced crude extract, (*2*) induced crude extract, (*3*) total soluble fraction taken after cell lysis by sonication, (*4*) insoluble fraction after sonication. *Red stars* indicate the 25 kDa fusion protein

**Fig. 3:**
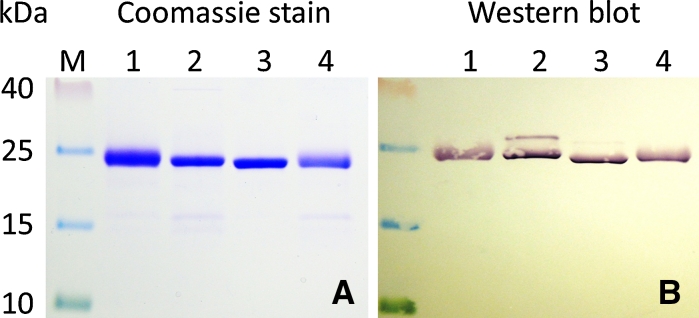
Comparison of Ni–NTA purified proTHI-TRX fusion proteins from strain C3030. **a** Coomassie Brilliant Blue staining. **b** Western blot with anti-His tag antibody. Each well contained 3 μg protein. (M) Prestained protein marker, (*1*) proTHI2.1-TRX, (*2*) proTHI2.2-TRX, (*3*) proTHI2.3-TRX, (*4*) proTHI2.4-TRX

The cytoplasm of *E. coli* is not well suited for the production of proteins containing disulfide bridges. However, *E. coli* strains with an oxidizing cytoplasm have been reported that result in high levels of functional proteins with disulfide bridges. We therefore cloned the expression plasmids for all four fusion proteins into the SHuffle strain C3030 (New England Biolabs) in which the lethal phenotype of lacking the *gor* and *trxB* reductases is suppressed by a mutation in the peroxidase *ahpC* (Faulkner et al. 2008) thus providing an oxidising environment in the cytoplasm. Additionally, expression of periplasmic disulfide bond isomerase (DsbC) in the cytoplasm of these strains is supposed to enhance proper disulfide bond formation (Maskos et al. 2003). We induced the expression with IPTG and separated the crude protein extract into soluble and insoluble proteins by centrifugation (Fig. 2). Again, only a small part of the proTHI2.2 and proTHI2.4 fusion protein was soluble. However, the fraction of soluble fusion protein was larger than that of the Rosetta strain (Supplementary Fig. 5). The amount of fusion protein that could be produced from the *SHuffle strain* C3030 is shown in Table 1 and was higher than obtained with Rosetta(DE3)pLysS. Western blotting of the purified fusion proteins (Fig. 3) showed less contaminating proteins than with the Rosetta(DE3)pLysS strain (Supplementary Fig. 6). Only the proTHI2.2 fusion protein showed a prominent extra band on the Western blot which was a few kDa larger than the fusion protein.

### TEV digestion of the fusion proteins

**Fig. 4:**
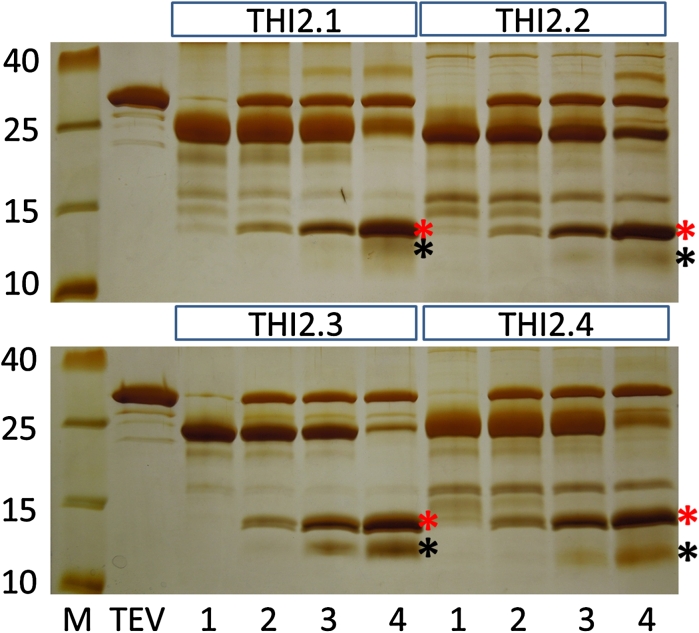
Silver stained tricine/SDS gel showing proTHI-TRX fusion proteins digested with TEV protease. Equal amounts (3.5 ug) of proThi-TRX was loaded. (M) Prestained protein marker, (TP) TEV protease, (*1*) undigested fusion protein, (*2*) fusion protein fraction digested with TEV protease, (*3*) fusion protein digested with TEV protease in the presence of glutathione (0.3 mM oxidized/3 mM reduced), (*4*) fusion proteins digested with TEV protease in the presence of 1 mM DTT. *Red stars* show the TRX protein and black stars show the thionin proproteins

All fusion proteins produced with the pETtrx_1a vector were digested with TEV protease. Since the thionin proproteins contained disulfide bridges, it was advantageous not to use DTT during TEV digestion; however, under these conditions all fusion proteins were poorly digested (Fig. 4). Inclusion of glutathione (0.3 mM oxidized/3 mM reduced) gave better digestion although still not complete with less than half of the fusion protein digested. Addition of DTT gave better results than glutathione with only a small amount of undigested fusion protein remaining.

### Antibacterial activity of fusion proteins

THI2.1 has *in planta* antifungal activity (Epple et al. 1997) and conditioned medium from cells expressing THI2.1 showed in vitro antimicrobial activity (Loeza-Ángeles et al. 2008). For several other thionins, antimicrobial activity in vitro has also been reported (Terras et al. 1993). Nothing is known yet about a possible antimicrobial activity of any thionin proprotein and thus it is also not known if the fusion proteins might have any activity. Antimicrobial activity of fusion proteins between TRX and AMPs has been reported (Wu et al. 2011) and could therefore not be excluded and might have been the reason that proTHI2.2 and proTHI2.4 fusion proteins were expressed rather poorly. We, therefore, tested the antibacterial activity of all TRX fusion proteins in vitro against the *E. coli* Rosetta strain. As shown in Supplementary Fig. 7, TRX itself did not inhibit the growth of *E. coli* at the concentrations tested (up to 100 ug/ml). However, high concentrations of proTHI-TRX fusion proteins showed clearly inhibitory activity especially after 8 h. The strongest activity was found for proTHI2.3-TRX which was inhibitory down to 25 μg/ml while the other thionin proprotein TRX fusions were only active at around 50 μg/ml.

## Discussion

Anti-microbial peptides (AMP) are difficult targets for heterologous expression in microbial hosts due to their intrinsic antibacterial and/or antifungal activity (Li 2009). In addition, the majority of plant AMPs contain disulfide bridges that are only formed under oxidizing conditions. In *E. coli*, the most popular microbial expression host, disulfide bridges are usually formed in the periplasm while the cytoplasm has reducing activity (reviewed by Salinas et al. 2011). To reduce the toxic activities of expressed proteins and/or to increase their solubility, a variety of different fusion partners have been used (reviewed by Terpe 2003). One fusion partner that has repeatedly been shown to enhance the solubility of fusion proteins is MBP. However, using MBP as a fusion partner in the vector pJOE-SP-MC, a derivative of the vector pJOE4905.1 (Motejadded and Altenbuchner 2009), we were unable to express the four *Arabidopsis* thionin proproteins as fusion proteins at useful levels in the periplasm. A reason for this might have been a proteolytic processing of the fusion proteins in the periplasm since the size of the fusion proteins was lower than expected. Another reason could have been that the fusion proteins were not properly secreted because they were misfolded and deposited in inclusion bodies, as the pellet that remained after osmotic shock contained the majority of the fusion proteins.

This result was somewhat surprising since we had produced THI2.1 proprotein before as a fusion with two protein As derived ZZ domains using the vector pExSecl (Epple et al. 1997). A serine protease inhibitor, turkey ovomucoid third domain, was similarly produced as a fusion with two IgG-binding Z domains (Lu et al. 1997). Other disulfide bridge-containing peptides have also been produced with other fusion partners in the periplasm in soluble form. For instance, the spider neurotoxic peptide Huwentoxin-I was expressed in the *E. coli* periplasm as a fusion with DsbC (Meng et al. 2011).

Since periplasmic expression did not produce undegraded fusion proteins, we tested cytoplasmic expression using two different *E. coli* strains, Rosetta(DE3)pLysS and C3030. The presence of DsbC inside the cytoplasm in the C3030 strain should allow better expression of proteins containing disulfide bridges. In all cases, proteins corresponding to the expected size of the fusion proteins were detected after induction; however, only part of it was soluble, sometimes only a small part, indicating that most of the foreign protein was deposited in inclusion bodies. The proTHI2.4 fusion protein especially could only be expressed at a rather low level. The reason for the different effectiveness in producing the thionin proproteins was not clear and we therefore tested the activity of all fusion proteins against *E. coli* because we reasoned that different toxicity of the fusion proteins could lead to differences in expression level. Indeed, we found significant activity of the proTHI fusion proteins on the growth of *E. coli* at higher concentrations. However, the strongest activity was found for the proTHI2.3-TRX fusion protein, indicating that toxic effects might not be the primary factor determining the expression level. On the other hand, we cannot exclude that the proTHI-TRX fusion proteins might have a different effect if produced inside the cell. Another difference between proTHI2.4 and the other proproteins is the pI which is strongly acidic (4.41) while the other three thionin proproteins have a pI above 7 (Table S3). Thus, the reason for the different efficiency in expressing the thionin proprotein fusion proteins remains unclear at the moment.

The TRX fusion proteins contained a TEV recognition site to release the thionin proproteins. It was originally assumed that Gly or Ser are required in the P1′ position of the TEV protease recognition site (Dougherty et al. 1988). The thionin proproteins have Lys or Asn at the *N*-terminus which would have required adding an additional amino acid, something that we do not accept for AMPs or their proproteins because it is not known if such an additional amino acid would have an effect on the activity. However, it has been shown that the requirement for Gly or Ser in the P1′-position is not that strict and that almost any amino acid is to some degree accepted (Kapust et al. 2002).

We therefore designed the TEV recognition sequence in such a way that the *N*-terminal amino acid of the thionin proprotein would be in the P1′-position of theTEV recognition site. Indeed, digestion of the TRX fusion proteins with TEV protease was successful for all four proproteins, although it was necessary to include DTT or glutathione. Inclusion of glutathione (0.3 mM oxidized/3 mM reduced) resulted in almost half of the fusion protein being digested. This was less than obtained with 1 mmol DTT; however, inclusion of DTT might lead to an opening of disulfide bridges and an inactivation of the proproteins. Thus, the production of proTHI-TRX fusion proteins and digestion with TEV protease would allow us to produce enough bona fide proproteins for further studies.

## Conclusions

Periplasmic expression seems to be the natural choice for the production of disulfide bridge containing proteins in *E. coli*. However, we have shown here that in case of thionin proproteins the expression as TRX fusion proteins in the cytoplasm of the *E. coli* strain C3030 was the method of choice.

## Electronic supplementary material

Below is the link to the electronic supplementary material.

Supplementary material 1 (DOCX 5801 kb)
