# Supplementary material for: Comparison of periplasmic and intracellular expression of Arabidopsis thionin proproteins in E. coli
Source: Biotechnol Lett. 2013 Mar 21;35(7):1085–91. doi: 10.1007/s10529-013-1180-z (PMC3677976; doi:10.1007/s10529-013-1180-z)
Supplement: Supplementary file 1 — Supplementary material 1 (DOCX 5801 kb) [file 10529_2013_1180_MOESM1_ESM.docx]

**Supplementary Material**

**Supplementary Methods**

*Cloning of pJOE-SP-MCS*

We modified the vector pJOE4905.1 for periplasmic expression by introducing the MBP R2 mutant signal peptide (Fikes et al. 1987) which can mediate MBP export to the periplasm with essentially the same efficiency as the wild-type signal peptide (Bankaitis et al. 1984). For cloning of this signal peptide, pJOE4905.1 was digested with NdeI which cuts at the start site of the *MalE* gene and Pfl23II which is 299 base pairs upstream of the start codon. This fragment was then replaced with one containing the R2 signal peptide and the beginning of the *MalE* gene as follows: The oligo MalESP was amplified by PCR using primers MalESPfor (introducing an *NdeI* site) and MalESPMrev. The beginning of the *MalE* gene was amplified with primers MalESPMfor and MalErev. Signal peptide and MalE fragment were fused by overlapping PCR using border primers (MalESPfor and MalErev). The final fragment was digested with NdeI and Pfl23II and subsequently cloned in pJOE4905.1 digested with the same enzymes to generate pJOE-SP which was confirmed by sequencing. A multiple cloning site at the end of the SUMO sequence was then introduced as follows: The oligos pJOEAadap3 and pJOEAadap4 were mixed in equal amounts, heat treated at 95°C for 15 min, and cooled down to form the polylinker adapter which was ligated to plasmid pJOE-SP digested with SspDI and BsrGI*.* This produced the final vector pJOE-SP-MCS. The region of the vector containing the signal peptide was confirmed by sequencing and the deduced sequence of the MalE fusion plus polylinker is provided in Fig. S2.

*Cloning of thionin proproteins in pJOE-SP-MCS*

Coding regions for thionin proproteins from *Arabidopsis thaliana* were amplified with primer pairs introducing a blunt end at the beginning of the thionin sequence (DraI or HpaI) supplied by the forward primer and a BamHI site behind the stop codon supplied by the reverse primer as shown in Table 2. These fragments were digested with DraI/HpaI and BamHI and ligated to pJOE-SP-MCS digested with SfoI and BamHI. This fuses SUMO and the thionin proproteins in frame. All constructs were confirmed by sequencing.

*Cloning of proproteins in pETtrx-1a*

The *TRX* gene was amplified with primers pETtrxfor1 and pETtrxTEVrev using pETtrx-1a as a template for 15 cycles. The thionin proproteins were also amplified by PCR for 15 cycles using specific forward primers which introduced a part of the TEV coding sequence before the start of the proprotein sequence and reverse primers introducing a BamHI site behind the stop codon. An overlapping PCR with pETtrxfor2 and the specific reverse primer joined the *TRX* sequence including the TEV site with the different proproteins. This PCR fragment was digested with XbaI and BamHI and cloned into the vector part of pETtrx-1A digested with the same enzymes. This fuses the TEV recognition sequence and the thionin proproteins in frame without any extra amino acids. Confirmation of the constructs was done by sequencing.

*Expression of MBP-SUMO fusion proteins and purification from the periplasm*

For expression from the pJOE vectors we used the *E. coli* strain Rosetta. Cells were grown in 1 l LB medium with 100 mg ampicillin/l and 34 mg/l chloromphenicol at 210 rpm and 37°C. When the cell density reached an OD_600_ of 0.6, the expression of fusion proteins was initiated by adding the inducer L-rhamnose (Molekula, UK) to a concentration of 2 g/l and growth was continued overnight at 28°C. Cells were harvested by centrifugation in a Sorvall RC 6+ centrifuge (Thermo Scientific) at 11250 g, 4°C for 15 min and resuspended in 20 ml osmotic shock buffer (300 mM Tris–HCl, 20% Sucrose; pH 8.1) and incubated at room temperature for 10 min followed by centrifugation in an Eppendorf Centrifuge 5418R(Eppendorf, Hamburg, Germany)at 8400 g at 4°C for 5 min. The pellet was incubated on ice for 10 min and then resuspended in cold 5 mM MgSO_4_ by vortexing gently. The total soluble periplasmic fraction (supernatant) was collected by centrifugation in an Eppendorf Centrifuge 5418R (Eppendorf, Hamburg, Germany) at 16800 g, 4°C for 10 min and lyophilized. The lyophilized protein was dissolved in Ni-NTA binding buffer (20 mM Na_2_HPO_4_, 400 mM NaCl, 20 mM imidazole; pH 7.4) and 450 µl Ni-NTA resin (Qiagen, Hilden, Germany) was added to the protein and incubated for half an hour with shaking at 4°C.The resin bound fusion protein was collected by centrifugation in an Eppendorf Centrifuge 5418R (Eppendorf, Hamburg, Germany) at 3600 g at 4°C for 5 min and was applied to a column (Vernet et al. 2011). The column was washed 10 times with one column volume of binding buffer and eluted three times with 500 μl elution buffer (20 mM Na_2_HPO_4_, 400 mM NaCl, 500 mM imidazole; pH 7.4).

*Expression of TRX-TEV fusion proteins and purification from the cytoplasm*

The modified TRX vectors containing thionin proproteins were transformed into chemical competent cells of the Rosetta(DE3)pLysS strain of *E. coli*. Transformed cells were selected on LB agar media plates containing 100 mg/l kanamycin and 34 mg/l chloramphenicol. A single colony was cultured in LB broth medium until it reached an OD_600_ of 0.5. The culture was then induced with 1 mM isopropyl-β-D-thiogalactoside (IPTG). After overnight growth at 20°C, the cells were pelleted by centrifugation in a Sorvall RC 6+ centrifuge (Thermo Scientific) at 10000 g at 4°C for 15 min. The cells were resuspended in binding buffer and sonicated on ice. Insoluble debris was removed by centrifugation in a Sorvall RC 6+ centrifuge (Thermo Scientific) at 45000 g for 20 min at 4°C. All subsequent purification procedures were carried out as described before except that the binding buffer contained 50 mM imidazole.

*Supplemental references*

Bankaitis VA, Rasmussen BA, Bassford PJ (1984) Intragenic Suppressor Mutations That Restore Export of Maltose Binding-Protein with a Truncated Signal Peptide. Cell 37: 243-252

Vernet E, Kotzsch A, Voldborg B, Sundstrom M (2011) Screening of genetic parameters for soluble protein expression in Escherichia coli. Protein Expr Purif 77: 104-111

**Supplementary Table 1**

E. coli strains and plasmids/vectors

| Vector | *E.coli* strain used for protein Expression | Localization in *E.coli* | Promoter Induction |
| --- | --- | --- | --- |
| pJOE-SP-MCS | Rosetta(DE3)plysS | Periplasm | L-rhamnose  2 g/L |
| pETtrx-1a | Rosetta(DE3)plysS, SHuffle strain C3030 | Cytoplasm | IPTG  1 mM |

**Supplementary Table 2**

Primers used in this work (restriction sites are underlined)

| Primer | Sequence | Amplification |
| --- | --- | --- |
| MalESPMfor | 5´TGCGCTGGCGAAAACTGAAG3´ | MalE region |
| MalErev | 5´GTTCGGCAGCAGATCTTTGT3 | MalE region |
| MalESPfor | 5´TATACATATGAAAATCAAAACTGG3´ | Signal Peptide |
| MalESPMrev | 5´CTTCAGTTTTCGCCAGCGCA3´ | Signal Peptide |
| MalESP | 5´CGCCAGCGCAGACGCAGAGAACATCGCCAGGATCAGCGCGCCAGTTTTGATT3´ | Signal peptide template |
| pJOEAadap3 | 5´GCGCCAAGTTTAAACTGGATCCT3´ | Polylinker |
| pJOEAadap4 | 5´GTACAGGATCCAGTTTAAACTTG3´ | Polylinker |
| pJOEfor | 5´CGAATTCAGGCGCTTTTTAG3´ | Sequencing primer |
| pJOErev | 5´CGCTTCTGCGTTCTGATTTA3´ | Sequencing primer |
| pETtrxfor2 | 5´TCCCGCGAAATTAATACGAC3´ | TRX fragment |
| pETtrxrev | 5´GGTGGTGGTGCTCGAGTG3´ | TRX fragment |
| pETtrxfor1 | 5´GTCCGGCGTAGAGGATCG3´ | TRX fragment |
| pETtrxTEVrev | 5´CTGAAAATAAAGATTCTCAGA3´ | TEV insertion |
| endTrxrev | 5´CTGCTGTTCAAAAACGGTGA3´ | Sequencing Primer |
| pETrev | 5´CCCCAAGGGGTTATGCTAGT3´ | Sequencing Primer |
| Thi2.1forTEV | 5´CTTTATTTTCAGAAGATCTGCTGCCCTTC3´ | proThi2.1 in TRX vector |
| Thi2.2forTEV | 5´CTTTATTTTCAGAAAATCTGCTGCCCTAC3´ | proThi2.2 in TRX vector |
| Thi2.3forTEV | 5´CTTTATTTTCAGAAAACTTGCTGCCCGAG3´ | proThi2.3 in TRX vector |
| Thi2.4forTEV | 5´CTTTATTTTCAGAATATTTGCTGCCCGTC3´ | proThi2.4 in TRX vector |
| THI2.1THforDra | 5´GAATTTAAAATCTGCTGCCCTTCC3´ | proThi2.1 in pJOE-SP-MCS vector |
| THI2.2THforDra | 5´AGAATTTAAAATCTGCTGTCCTAC3´ | proThi2.2 in pJOE-SP-MCS vector |
| THI2.3THforDra | 5´GCATTTAAAACTTGCTGCCCGAGCC3´ | proThi2.3 in pJOE-SP-MCS vector |
| THI2.4THforHpa | 5´TGACGTTAACATTTGCTGTCCGTC3´ | proThi2.4 in pJOE-SP-MCS vector |
| Thi2.1RevBam | 5´GAGTTTGGGATCCTTTAGTAAGGA3´ | proThi2.1 in pJOE-SP-MCS & TRX vector |
| Thi2.2THrevBam | 5´TAAAGGATCCTGTTTAGGCACTTTTAAC3´ | proThi2.3 in pJOE-SP-MCS & TRX vector |
| Thi2.3ThrevBam | 5´AGAGGATCCGAGACTTTAGTAAGGA3´ | proThi2.3 in pJOE-SP-MCS & TRX vector |
| Thi2.4revBam | 5´ATATGGATCCCTACGCATTTTCAACTGCAT3´ | proThi2.4 in pJOE-SP-MCS & TRX vector |

**Supplementary Table 3**

Isoelectric point of thionin peptides

|  | Thionin | Acidic domain | Proprotein |
| --- | --- | --- | --- |
| THI2.1 | 9.43 | 4.75 | 8.30 |
| THI2.2 | 8.86 | 4.44 | 7.56 |
| THI2.3 | 4.66 | 8.63 | 7.56 |
| THI2.4 | 7.77 | 4.06 | 4.41 |

**Supplementary Table 4**

Calculated Mol weight (kDa) of MBP fusion proteins

|  | SP | MBP | HIS-tag | SUMO | Fusion | Thioninpro | Total with SP | Total without SP |
| --- | --- | --- | --- | --- | --- | --- | --- | --- |
| THI2.1 | 1.94 | 42.89 | 0.84 | 10.26 | 55.93 | 11.71 | 67.64 | **65.70** |
| THI2.2 | 1.94 | 42.89 | 0.84 | 10.26 | 55.93 | 11.62 | 67.55 | **65.61** |
| THI2.3 | 1.94 | 42.89 | 0.84 | 10.26 | 55.93 | 11.53 | 67.46 | **65.52** |
| THI 2.4 | 1.94 | 42.89 | 0.84 | 10.26 | 55.93 | 11.53 | 67.46 | **65.52** |

**Supplementary Table 5**

Calculated Mol weight (kDa) of TRX fusion proteins

|  | HIS-tag | TRX+TEV | Fusion | Thioninpro | Total |
| --- | --- | --- | --- | --- | --- |
| THI2.1 | 0.84 | 13.16 | 14 | 11.71 | **25.71** |
| THI2.2 | 0.84 | 13.16 | 14 | 11.62 | **25.62** |
| THI2.3 | 0.84 | 13.16 | 14 | 11.53 | **25.53** |
| THI2.4 | 0.84 | 13.16 | 14 | 11.53 | **25.53** |

**THI2.1 KICCPSNQARNGYSVCRIRFSKG-RCMQVSGCQNS---DTCPRGWVN▼**

**THI2.2 KICCPTKDADRSVYFVCMLSVSSQFYCLLKSKCKNTSQTICPPGYTN▼**

**THI2.3 KTCCPSQSTRKEFEDCISEGNLQILCSAESGCRD-TYVGYCPSGFPY▼**

**THI2.4 NICCPSIQARTFYNACLFAVGSPSSCIRNSSCLD-ISESTCPRGYTN▼**

**AILENSADATNEHCKLGCETSVCGAMNTLQNSDASEIVNGASEQCAKGCSIFCTKSYVVPPGPPKLL**

**DILENSGDAVNEYCKLGCASSVCGALTTLQNFDTSKVLSEAVEQCTKACSSVCTGGSTAAVKSA GSLTNSGDVVNVYCKLGCVSSLCGALTSLQKLDTSGKVNVAVERCTKACSTICTKGSKTAVETV**

**DILENTGDAVTEYCKLGCVSSVCGALTILQNSDASEIVNGEVEKCTMACSTVCTKGSMNAVENA**

**Supplementary Fig. 1** Sequences of Arabidopsis thionin proproteins.

Basic amino acids marked blue, acidic amino acids marked red.

**Supplementary Fig. 2** Sequence of the vector pJOE-SP-MCS. The text highlighted yellow shows the inserted signal peptide while the hexahistidine tag is highlighted green. Red arrow between G and A amino acids is the cleavage site of SUMO protease. The BamHI site (grey) present in the polylinker (blue) was used for cloning of proproteins in this plasmid.

**
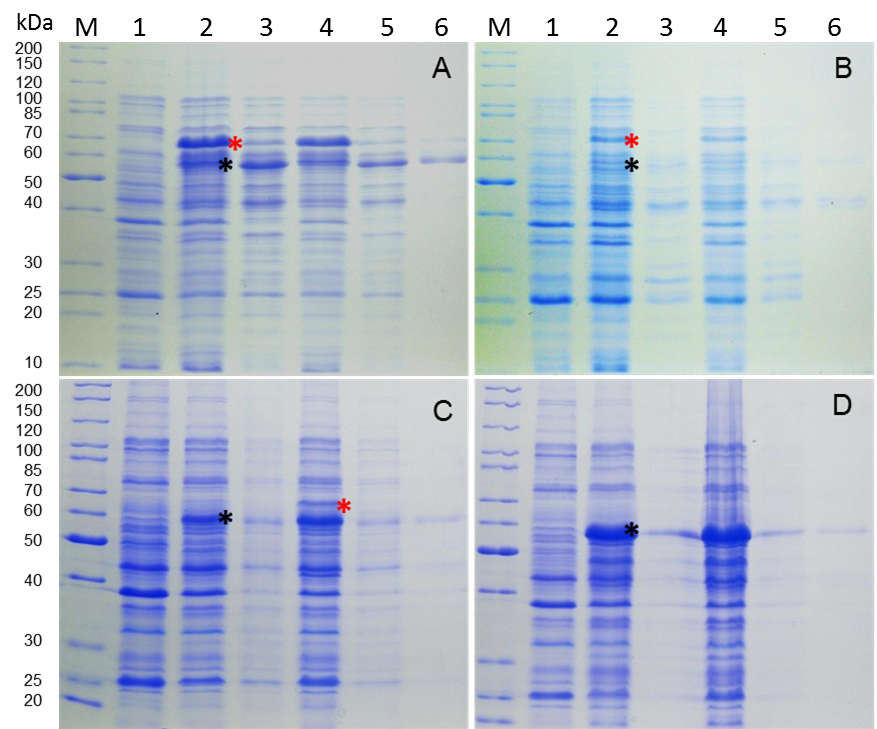
**

**Supplementary Fig. 3** Periplamic MBP fusion protein expression in *E. coli* Rosetta. (A) THI2.3-MBP fusion protein; (B) THI2.2-MBP fusion Protein; (C) THI2.3-MBP fusion Protein; (D) THI2.4-MBP fusion protein. Pellet from 1 ml cell culture was dissolved in 100 µl of sample buffer and 10 µl was loaded on a PAGE gel; equivalent amounts were loaded for the other fractions. (M) Protein Marker; (1) uniduced crude extract; (2) rhamnose induced crude extract; (3) total periplasmic extract obtained through osmotic shock; (4) Left over fraction (unsoluble) after isolating the total periplasmic fraction; (5) flow through from Ni-NTA column; (6) Ni-NTA column purified THI-MBP. Red stars indicate the 65 kDa fusion protein and black stars indicate a 58 kDa protein.

For proTHI2.1, proTHI2.3 and proTHI2.4 strong extra bands and a faint band in case of proTHI2.2 of a smaller size (58 kDa) than expected (65 kDa, Table S2) were found in the total cell extract after induction but not without induction (lanes 1 and 2, black stars). In case of proTHI2.1 there was a strong band after induction of the expected size of about 65 kDa. The periplasmic fractions (lanes 3) showed proteins of the expected (65 kDa, Table S2) for proTHI2.1 only and a very faint band for proTHI2.2. In case of proTHI2.1, the band of 58 kDa was also clearly visible and the band for the 65 kDa protein was much weaker than in the total protein fraction. However, the majority of the fusion proteins remained in the insoluble fraction (lanes 4). In case of proTHI2.3 the insoluble fraction showed a clear band for the expected fusion protein (red star) which was not detected in the periplasmic fraction. From the periplasmic protein extract the fusion proteins were enriched by Ni-NTA affinity chromatography. The eluted proteins (lanes 6) contained clearly visible amounts of the expected fusion protein only in case of proTHI2.1; however, the 58 kDa band was much more prominent. The affinity chromatography could only recover part of the fusion proteins as the fusion proteins were also detected in the flow through of the column (lanes 5).

**
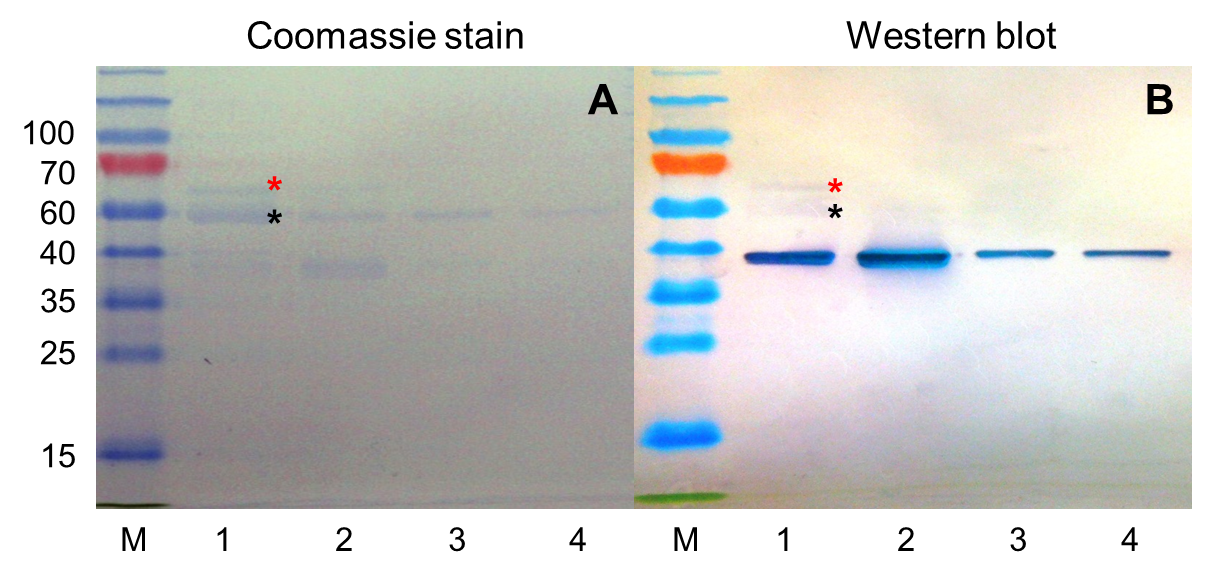
**

**Supplementary Fig. 4** Comparison of Ni-NTA purified proTHI-MBP fusion proteins from Rosetta strain, (A) Commassie brilliant blue staining. (B) Western blot with anti-His tag antibody. In each slot 3 µg of protein was loaded, (M) Prestained Protein Marker; (1) proTHI2.1-TRX; (2) proTHI2.2-TRX; (3) proTHI2.3-TRX; (4) proTHI2.4-TRX. Red stars indicate the fusion protein and black stars indicate 58 kDa bands


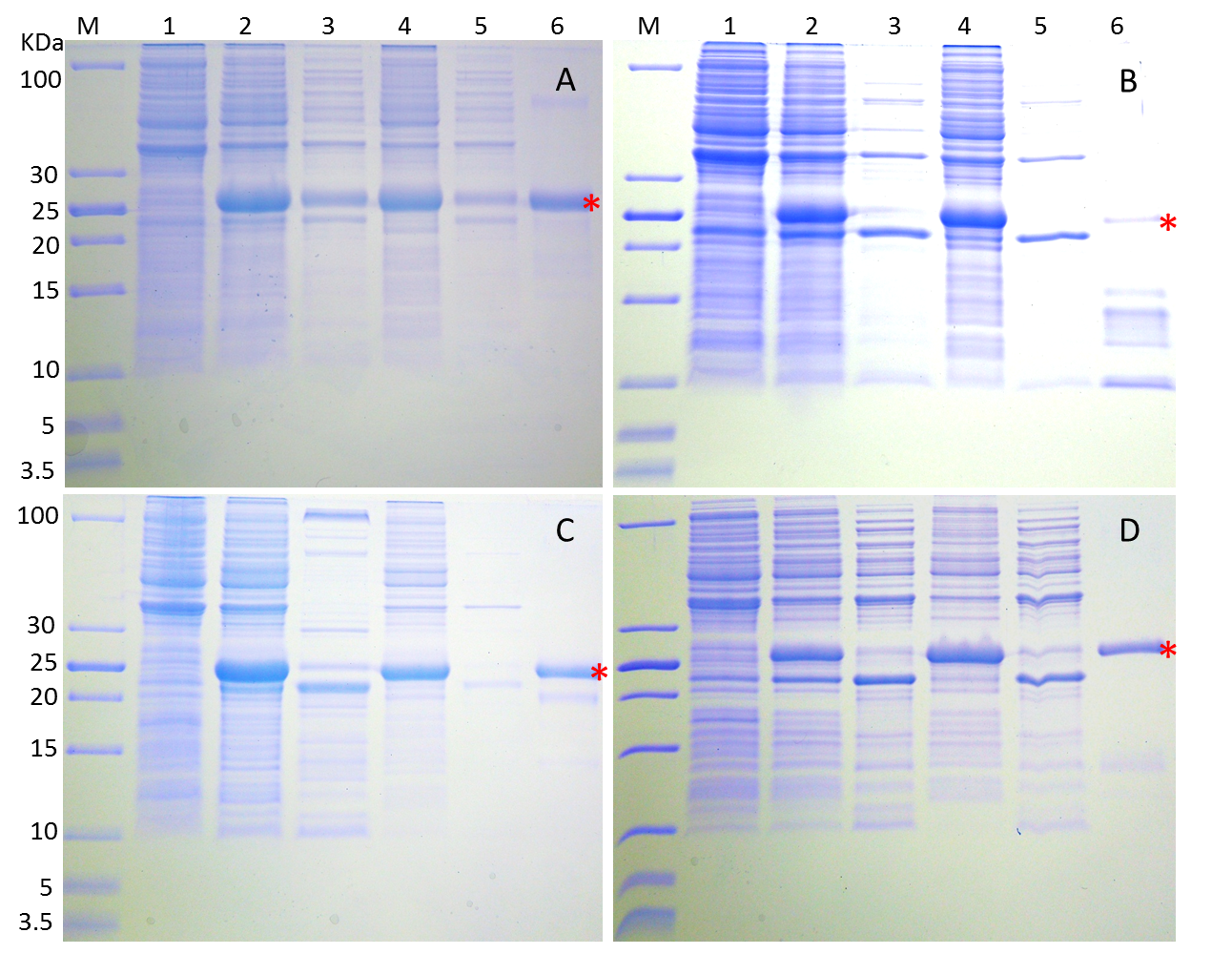


**Supplementary Fig. 5** Cytoplamic expression of proTHI-TRX fusion proteins in the Rosetta(DE3) pLysS strain. (A) proTHI2.1-TRX fusion protein; (B) proTHI2.2-TRX fusion protein; (C) proTHI2.3-TRX fusion protein; (D) proTHI2.4-TRX fusion protein. (M) Protein Marker; (1) uniduced crude extract; (2) IPTG induced crude extract; (3) total soluble fraction after sonication; (4) insoluble fraction after sonication ; (5) flow through from Ni-NTA column; (6) Ni-NTA column purified proTHI-TRX fusion proteins. Red stars indicate the 25 kDa fusion protein.


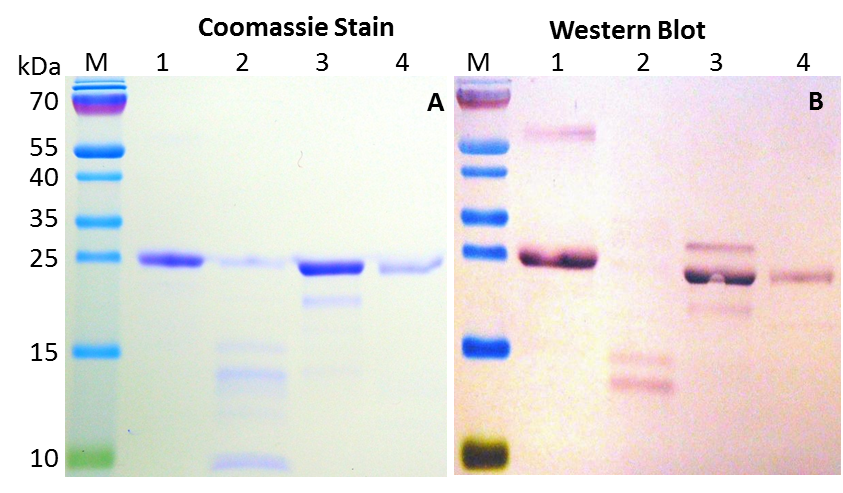


**Supplementary Fig. 6** Comparison of Ni-NTA purified proTHI-TRX fusion proteins from strain Rosetta(pLysS)DE3, (A) Coomassie brilliant blue staining. (B) Western blot with anti-His tag antibody. In each slot 3 µg of protein was loaded, (M) Prestained Protein Marker; (1) proTHI2.1-TRX; (2) proTHI2.2-TRX; (3) proTHI2.3-TRX; (4) proTHI2.4-TRX.


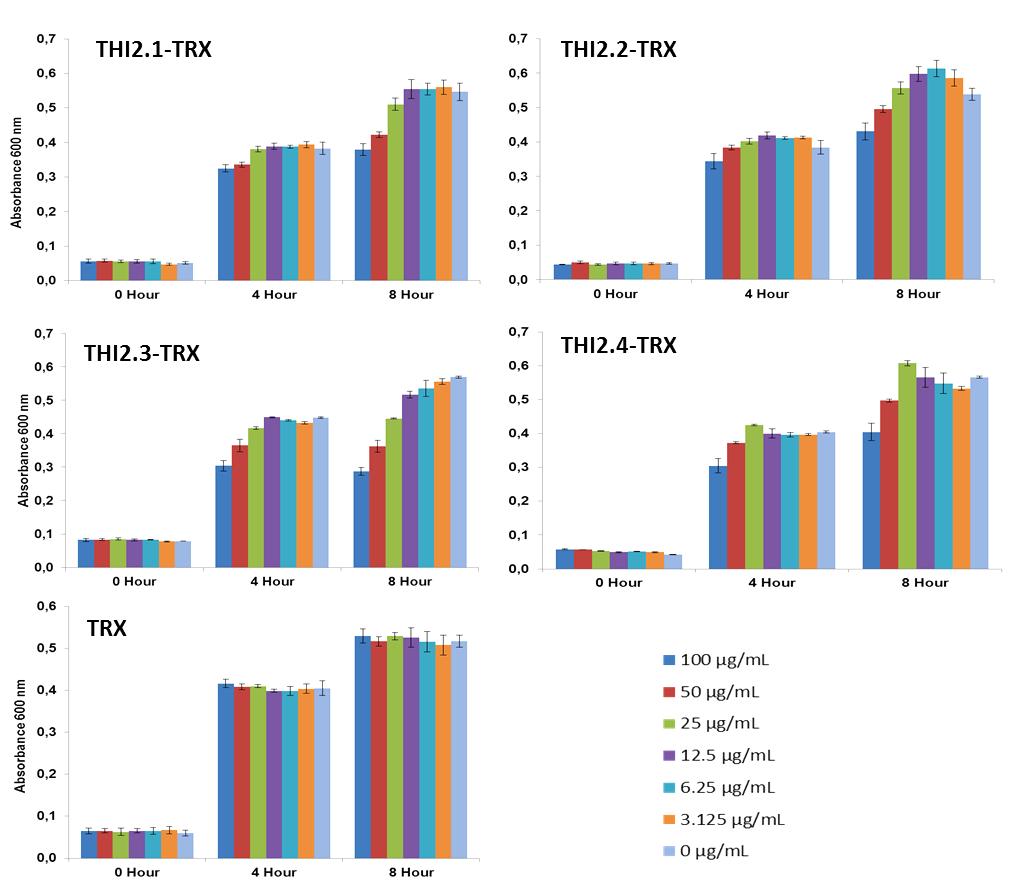


**Supplementary Fig. 7** The effect of proTHI-TRX on growth of the *E. coli* Rosetta strain. OD_600_ was measured at 0, 4, and 8 hours. Fusion proteins were diluted in water and TRX was used as control. Fifty µl of the peptide were tested against 150 μl of culture at final concentrations of 100 μg/ml - 3.125 μg/ml. Data for three replicates are shown.
